# Predicting sumoylation sites using support vector machines based on various sequence features, conformational flexibility and disorder

**DOI:** 10.1186/1471-2164-15-S9-S18

**Published:** 2014-12-08

**Authors:** Ahmet Sinan Yavuz, Osman Ugur Sezerman

**Affiliations:** 1 Biological Sciences and Bioengineering Program, Faculty of Engineering and Natural Sciences, Sabanci University, 34956, Istanbul, Turkey

**Keywords:** Sumoylation, SUMO, machine learning, support vector machines, post-translational modification

## Abstract

**Background:**

Sumoylation, which is a reversible and dynamic post-translational modification, is one of the vital processes in a cell. Before a protein matures to perform its function, sumoylation may alter its localization, interactions, and possibly structural conformation. Abberations in protein sumoylation has been linked with a variety of disorders and developmental anomalies. Experimental approaches to identification of sumoylation sites may not be effective due to the dynamic nature of sumoylation, laborsome experiments and their cost. Therefore, computational approaches may guide experimental identification of sumoylation sites and provide insights for further understanding sumoylation mechanism.

**Results:**

In this paper, the effectiveness of using various sequence properties in predicting sumoylation sites was investigated with statistical analyses and machine learning approach employing support vector machines. These sequence properties were derived from windows of size 7 including position-specific amino acid composition, hydrophobicity, estimated sub-window volumes, predicted disorder, and conformational flexibility. 5-fold cross-validation results on experimentally identified sumoylation sites revealed that our method successfully predicts sumoylation sites with a Matthew's correlation coefficient, sensitivity, specificity, and accuracy equal to 0.66, 73%, 98%, and 97%, respectively. Additionally, we have showed that our method compares favorably to the existing prediction methods and basic regular expressions scanner.

**Conclusions:**

By using support vector machines, a new, robust method for sumoylation site prediction was introduced. Besides, the possible effects of predicted conformational flexibility and disorder on sumoylation site recognition were explored computationally for the first time to our knowledge as an additional parameter that could aid in sumoylation site prediction.

## Background

Small ubiquitin-like modifier (SUMO) proteins are small proteins with an approximate size of 10 kD. They have a high structural similarity with ubiquitin, while sharing up to only 20% sequence identity. In spite of this structural similarity, SUMO proteins have an unstructured stretch of 10-25 amino acids at their N termini, which is not seen in ubiquitin proteins [1]. SUMO proteins are expressed ubiquitously in the eukaryotic kingdom, and they show high evolutionary conservation rates [1]. Some of the organisms, such as *C. elegans *and *D. melanogaster*, have only one SUMO gene, while plants and vertebrae have multiple SUMO genes with different properties. Human genome has 4 distinct and slightly different SUMO paralogs, named SUMO1-4. Of these, SUMO1-3 can be found in almost every tissue in humans; however, SUMO4 has been mainly seen in kidneys, lymph nodes and spleen (reviewed in [1]). Additionally, proteomic studies showed that SUMO1 and SUMO2/3 have partially overlapping sets of target proteins. Of these identified target proteins, nearly one-third are putative transcription regulators while the other two-thirds are composed of signalling molecules, nuclear envelope proteins, and cell membrane proteins (reviewed in [2]).

SUMO proteins are inactive when they are synthesized and they become activated after the cleavage of a C terminal peptide (2-11 amino acids long) by sentrin-specific proteases, exposing an invariant Gly-Gly motif [1]. After this cleavage, SUMO proteins become ready for the sumoylation process and attachment to a target protein.

**Figure 1:**
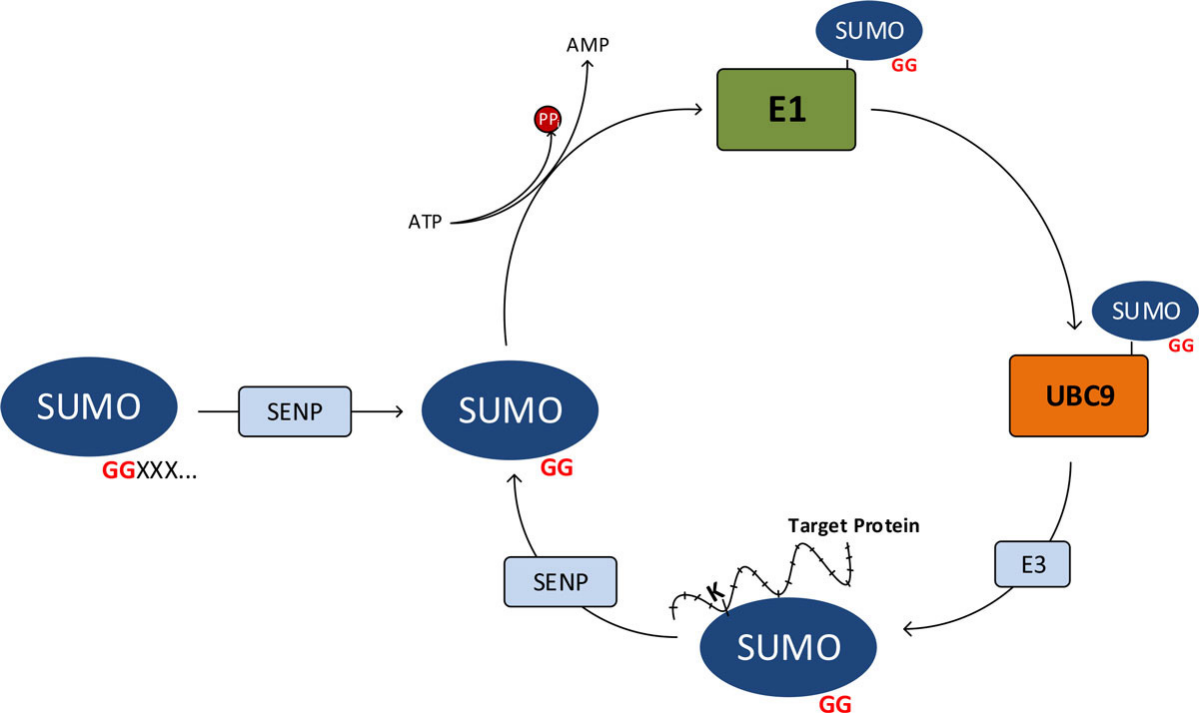
**The reversible sumoylation mechanism**. The sumoylation pathway starts with an immature SUMO protein that needs to be protealytically processed by SENPs to reveal its target binding site, an invariant Gly-Gly motif. The mature SUMO protein is then activated by E1 heterodimer in an ATP-dependent reaction. SUMO is then transferred to an E2 enzyme, UBC9, which is responsible for the recognition of target binding sites. After the recognition, the SUMO protein is transferred to a lysine residue in the target binding site. This process is generally assisted by an E3 ligase. Sumoylated sites can also act as substrates for SENPs, so the sumoylation can be reversed. This ensures the dynamic and reversible nature of sumoylation.

Sumoylation pathway involves a SUMO-protease enzyme, the activating enzyme E1, the conjugating enzyme E2 and the mediator enzyme E3 (Figure 1). UBC9 plays the key role in the pathway as it recognizes the SUMO binding motif, which generally has the consensus motif of ΨKxE/D (Ψ is a large aliphatic hydrophobic residue and × is any amino acid) [1]. Two main extensions of this motif has been proposed in literature (reviewed in [1]). The first one is phosphorylation-dependent sumoylation motif (PDSM), in which a phosphorylated serine and a proline residue is present after the conventional binding motif (ΨKxExxpSP). The second extended motif is the negatively charged amino-acid dependent sumoylation motif (NDSM). The common theme for both motifs is that the negative charge next to the basic SUMO consensus site enhances the sumoylation [1]. However, having one of these motifs is not a complete indicator of sumoylation. It has been argued that subcellular localization and/or appropriate sequence presentation may also be of significance in determination of a sumoylation site. Hence, accurate identification of structural context of the target protein becomes the main issue, as the structural context seems to dictate the sumoylation [2]. Additionally, it has been argued that other post-translational modifications may have effects on sumoylation, such as acetylation can act as a preparation step for sumoylation, which was the case for histone H4 [2].

Rapidly progressing research on sumoylation showed that sumoylation can alter the target's localization, intra/intermolecular interactions, and resulting roles in protein stability, protein-DNA binding activity, transcriptional regulation, sub-nuclear targeting, nucleocytoplasmic translocalization, chromosome segregation and various indispensable roles in mitosis [1-7]. In addition to these vital cellular processes, sumoylation is linked with ever increasing number of diseases, such as Alzheimer's, Parkinson's, Huntington's, Multiple Sclerosis and cancer (reviewed in [8-14]). However, identifying sumoylation sites experimentally is a labor-intensive process, which may also result in false negatives due to the reversible and dynamic nature of sumoylation. On the other hand, basic motif matching based computational methods are predestined with a certain classification accuracy, as approximately 26% (97/381) of the known cases do not contain the identified consensus motif (ΨKxE/D). Hence, developing a reliable *in silico *sumoylation prediction method that has a better accuracy based on sequence derived features, bears a significant importance for the understanding complex diseases, cellular processes and epigenetic mechanisms.

In order to overcome this challenge, various sophisticated methods have been introduced into literature. One of the earliest predictors of sumoylation was SUMOplot [15], which is mainly based on consensus motif matching and substitution of the consensus amino acid residues with amino acid residues that have similar hydrophobicity. Following SUMOplot, SUMOpre [16], a probabilistic model that optimizes the entropy of the motif, was published. Another method, SUMOsp [17] based on 2 algorithms originally introduced for prediction of phosphorylation sites, group based scoring (GPS) [18], and another statistical method named MotifX [19]. SUMOsp was updated to SUMOsp 2.0 a year later [20]. Meanwhile, Bauer et al. [21] has developed another method, based on sequence window representations with amino acid composition, evolutionary information, relative solvent accessibility and secondary structure. seeSUMO [22], a recently published method, was mainly based on random forest and SVM training of biological sequence features obtained from AAIndex and evolutionary information of sequence windows. Lastly, SUMOhydro [23] is mainly based on input feature vectors based on various representations, including, but not limited to, traditional binary encoding, the composition of k-spaced amino acid pairs (CKSAAP), position specific scoring matrix, six-letter, nine-letter, and 'hydrobinary' encoding. Among these methods, SUMOplot, SUMOsp 2.0, seeSUMO, and SUMOhydro have active web servers that can be used to predict sumoylation sites in a protein. Although these methods have comparable prediction performances, they are not sufficient for understanding the complete picture of sumoylation mechanism.

In this study, we have investigated efficacy of various physicochemical properties of lysine centered sequence windows in sumoylation site prediction. We have introduced the use of new properties, such as lysine conformational flexibility and disorder. The sumolated lysine should be flexible to interact with the SUMO protein and disorder information may shed light on the mechanism of interaction. This is because lysines in ordered structures may encounter some degree of difficulty in sumoylation depending on their structural location. We also used the position of the lysine with respect to the vicinity to either termini, as well as the previously used properties, such as hydrophobicity, amino acid volumes and direct sequence information. By using fine-tuned support vector machines (SVM) we have been able to achieve a robust performance displayed by 5-fold cross validation results with Matthew's correlation coefficient, sensitivity, specificity and accuracy of 0.66, 73%, 98%, 97%, respectively. Here we have presented the statistical analysis of sumoylated sites, details of this new method, its overall performance, and in depth benchmark experiments with 3 previously published methods and a simple consensus motif regular expressions scanner.

## Results

### Dataset and statistical analysis

The training set consisted of 267 positive sites conforming to the consensus motif, 90 positive sites (~25% of positive sites) not conforming to the consensus motif, 280 negative sites conforming to the consensus motif, and 7629 negative sites not conforming to the consensus motif (Table 1). Similarly, the test set was composed of 17 positive sites conforming to the consensus motif, 7 positive sites not conforming to the consensus motif, 22 negative sites conforming to the consensus motif and lastly, 488 negative sites not conforming to the consensus motif (Table 1).

**Table 1:** Dataset distribution.

	Training Set	Test Set	
	**Sites (%)**	**Sites (%)**	**TOTAL**

Positive Sites (Consensus)	267 (3.23)	17 (3.18)	284
Positive Sites (Non-consensus)	90 (1.09)	7 (1.31)	97
Negative Sites (Consensus)	280 (3.39)	22 (4.11)	302
Negative Sites (Non-consensus)	7629 (92.29)	488 (91.39)	8117
**Total**	8266 (100)	534 (100)	8800

A total of 381 experimentally verified sumoylation sites were divided into 4 categories. 357 of the positive sites formed the training set, in which 267 sites conformed to the consensus motif and 90 did not conform to the consensus motif. Remaining 24 positive sites formed the independent testing set, in which 17 conformed to the consensus motif and, 7 did not conformed to the consensus motif.

**Figure 2:**
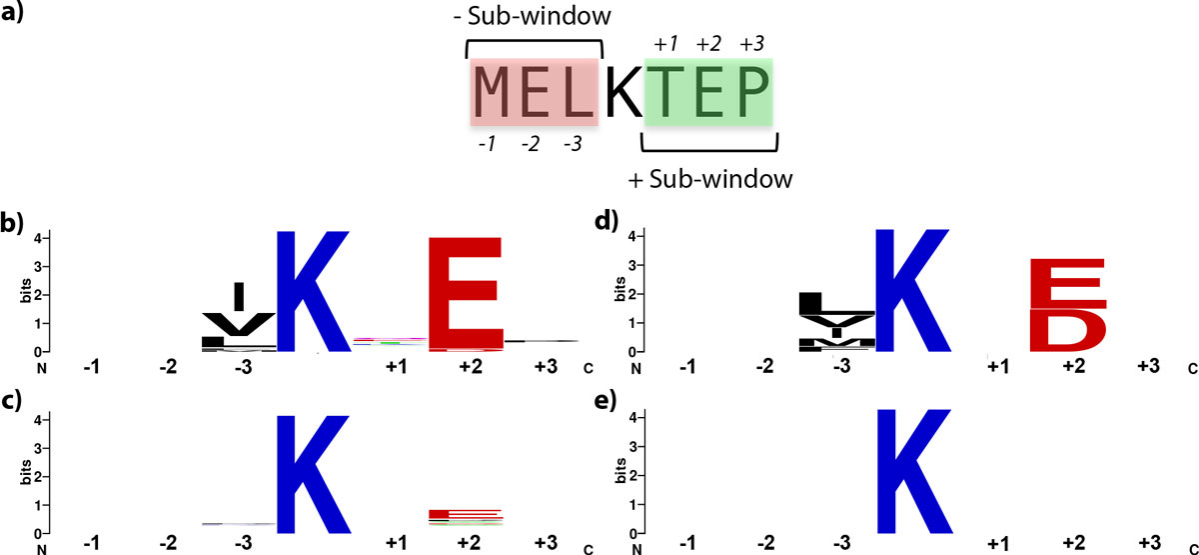
**Window position representation and comparison of sequence logos between positive and negative sites**. **a) **Position nomenclature that has been used throughout the study. Sequence windows divided into negative (-) and positive (+) sub-windows. In each subwindow, amino acids are numbered in an incrementing order. **b) **Sequence logo of positive consensus sites indicating preferences in amino acids in each window position. **c) **Sequence logo of positive non-consensus sites. **d) **Sequence logo of negative consensus sites. **e) **Sequence logo of negative non-consensus sites.

Most of the sumoylated sites are identified by the existence of the consensus motif ΨKxE/D (~75%). We have created sequence logos for observing amino acid distribution of consensus positive sites (Figure 2b), non-consensus positive sites (Figure 2c), consensus negative sites (Figure 2d), and non-consensus negative sites (Figure 2e). In consensus positive sites, central lysine residue predominantly flanked by glutamic acid residue in C-terminal side. On the other hand, in consensus negative sites, central lysine is almost equally flanked by glutamic acid or aspartic acid in C-terminal site. These slight differences have been shown to be insufficient to predict all the sumoylation cases. Therefore, there should be other factors facilitating SUMO binding to the target protein or more subtle differences in amino acid preferences that cannot be represented with sequence logos. In this section, we discuss statistical differences between sumoylated sequence windows and non-sumoylated sequence windows.

We have performed three different statistical comparisons: positive windows vs. negative windows, consensus positive windows vs. non-consensus positive windows, and consensus negative windows vs. non-consensus negative windows (see Methods for details). Separate chi-square tests of independence were performed for each amino acid presence in the each position of the sequence window (see Figure 2a for position naming for sequence windows). For the hydrophobicity and estimated volume, non-parametric Mann-Whitney U tests were performed. Out of 137 different statistical tests for the first strategy, 47 yielded significant results with p < 0.05. 37 out of 47 (~79%) statistically significant features were also retained in feature selection (Table 2). Among those, we have explored only a portion of statistically significant features in this section due to space limitations. The rest of the statististical testing results with their descriptive statistics, p-values, and their test statistics can be found in the additional file 1.

**Table 2:** Top 25 features selected using RELIEFF algorithm.

Rank	Feature	Merit Score	P-value	Adj. P-value	Significance
1	w+E_2	0.355474	0.00E+00	0.00E+00	*
2	Consensus	0.261813	0.00E+00	0.00E+00	*
3	wDE	0.164459	1.66E-41	3.23E-40	*
4	w+2_Hydro	0.160149	3.90E-83	1.33E-81	*
5	w-I_3	0.105916	1.18E-107	5.33E-106	*
6	w-3_Hydro	0.104835	8.12E-58	1.84E-56	*
7	wK	0.078651	1.33E-02	4.03E-02	*
8	w-V_3	0.075073	1.92E-58	5.22E-57	*
9	w-2_Hydro	0.057669	1.48E-02	4.37E-02	*
10	w+3_Hydro	0.056496	1.49E-01	2.93E-01	
11	w+1_Hydro	0.05232	7.13E-02	1.62E-01	
12	w-1_Hydro	0.051279	4.22E-02	9.89E-02	
13	w-L_3	0.051001	1.96E-03	7.00E-03	*
14	w+K_2	0.050248	1.70E-08	1.78E-07	*
15	w+P_2	0.045911	3.39E-02	8.23E-02	
16	w+P_3	0.043573	2.11E-25	3.58E-24	*
17	w-K_3	0.043208	4.33E-04	1.96E-03	*
18	Flexibility	0.042334	7.52E-07	7.31E-06	*
19	w+D_2	0.041784	7.14E-01	7.77E-01	
20	w-S_2	0.041097	2.95E-01	4.57E-01	
21	DisorderBinary	0.040666	7.27E-14	9.88E-13	*
22	w-E_3	0.039548	6.14E-05	3.79E-04	*
23	w-A_3	0.03804	3.04E-02	7.95E-02	
24	w-P_1	0.037893	4.46E-06	3.80E-05	*
25	w-E_2	0.035935	4.16E-01	5.74E-01	

93 out of 137 features has been selected based on 10-fold classification performance. Features are ranked using RELIEFF [26] algorithm, implemented in Weka [39]. Details of statistical testing can be found in Methods section. For assessing significance, adjusted p-value cutoff of 0.05 is used. Feature name explanations can be found in Methods section and Figure 2a.

The proportion of glutamic acid at one amino acid after lysine (w+E_2), a consensus motif feature, was 0.81 in positive windows, whereas it was 0.07 in negative windows. This difference in proportions was found to be significant, χ² (1, N = 8266) = 1998.27, p < 0.001. This result also can also be supported by sequence logos in Figure 2b-e.

A depletion of positive amino acid residues in particular positions of sequence windows has been observed in sumoylated cases. For instance, the proportion of an extra lysine located 2 residues after central lysine (w+K_2) was 0.01 in positive windows, and 0.09 in negative windows. The difference was found statistically significant, χ² (1, N = 8266) = 31.81, p < 0.001, with Yates' correction. Similarly, the proportion of an extra lysine residue presence just before the central lysine residue (w-K_3) was 0.03 in positive windows, whereas it was 0.09 in negative windows. This difference was found to be statistically significant as well, χ² (1, N = 8266) = 12.38, p = 0.002. Same depletion pattern was also observed for the arginine presence in exactly same locations in the sequence windows. The associated proportions were 0.01 in positive windows, and 0.06 in negative windows for arginine presence two residues after central lysine residue (w+R_2). This difference was found to be significant, χ² (1, N = 8266) = 14.59, p < 0.001, with Yates' correction. For the presence of arginine residue just before central lysine (w-R_3), the proportions were 0.01 in positive windows and 0.07 in negative windows. This proportion difference was also found to be significant, χ² (1, N = 8266) = 18.1, p < 0.001, with Yates' correction. A similar pattern can be seen for histidine presence in second position after central lysine (w+H_2) as well (proportions were 0 in positive sites, 0.03 in negative sites, χ² (1, N = 8266) = 6.49, p = 0.035, with Yates' correction).

An interesting result was the presence of proline at the end of the sequence window (w+P_3). The proportions were 0.19 in positive windows and 0.06 in negative windows. The difference of these proportions was found to be significant, χ² (1, N = 8266) = 108.48, p < 0.001. This position was argued to be important in controlling the acetylation-sumoylation switch on the lysine residue and it has been documented that acetylation is antagonizing the sumoylation (reviewed in [24,25]). In fact, an extension to the consensus motif was identified for cross-play between sumoylation and acetylation: ΨKxEP [24]. This result gives rise to two questions. The first one is what the proportion of shared targets between acetylation and sumoylation is. The second question is whether the presence of proline residue at this specific position also favors sumoylation. This can be due to some kind of structural similarity of acetylation and sumoylation enzymes as they may recognize the same lysine residue as a target.

Most striking differences we have observed were in flexibility and disorder. As they have never been systematically explored in sumoylation context, our findings may be of help in understanding the sumoylation process. The proportion of conformationally flexible central lysine residue was found to be 0.57 in positive windows, whereas it was 0.44 in negative windows. This difference in proportions was found to be significant, χ² (1, N = 8266) = 24.48, p < 0.001. Similarly, disorder (DisorderBinary) was found to be significantly different between positive and negative windows (proportions was 0.59, and 0.39, respectively), χ² (1, N = 8266) = 56, p < 0.001. These results may suggest that in order for sumoylation to occur the corresponding lysine residue should be flexible enough to be captured by sumoylation enzymes and SUMO binding. Another interesting result on disorder was proportional difference between consensus positive sites and non-consensus positive sites (the second comparison strategy). A chi-square test of independence was performed to determine how consensus and non-consensus positive sites differ from each other. Apart from various amino acid preferences, the proportion of disordered central lysine residues (DisorderBinary) was 0.64 in consensus positive sites, whereas it was 0.43 in non-consensus positive sites. This difference was found to be significant, χ² (1, N = 357) = 11.52, p = 0.007, which may suggest a mechanistic difference between consensus site sumoylation and non-consensus site sumoylation.

The third strategy resulted in several amino acid preferences on various positions of the sequence window. However, we have omitted the details of those for the sake of brevity and they can be found in the supplementary material. We would like to also note that statistical testing results should be taken with caution. Especially, some of the amino acid preferences may be declared significant only due to frequency differences, not to an underlying biological principle.

### Prediction performance and feature selection

**Figure 3:**
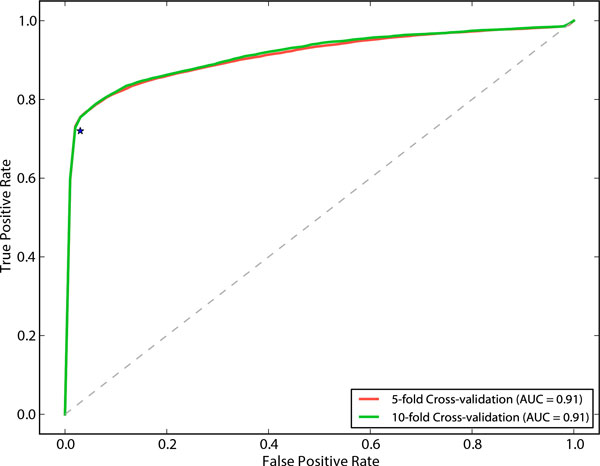
**Receiver operator characteristic (ROC) curves**. Average ROC for 25 repeats of 5-fold cross-validation and 10-fold cross validation. AUC is calculated using the average ROC curve. The dashed line represents a random classifier and the star indicates the performance of regular expressions scanner.

SUMOsu is a new method developed on an already existing dataset, with a new set of sequence based features, by employing support vector machines. In order to improve prediction performance; kernel, C-values, γ, and window size were optimized for this problem specifically (details can be found in the Methods section). Feature selection has resulted in 93 optimal features (~68%) from a total of 137 features. The ranked list of top 25 features with their statistical analysis results can be found in Table 2. The complete list of selected features is presented in additional file 2. Using optimized parameters and selected features, SUMOsu was able to identify sumoylated sites accurately. In order to test the stability of our method, we have implemented three evaluation strategies: self-consistency test, where training dataset was used as both training and test dataset, 10-fold cross-validation, and 5-fold cross-validation. Four different evaluation measures have been calculated for assessing performance of stated strategies: accuracy, specificity, sensitivity and Matthew's correlation coefficient (MCC) (see the Performance evaluation subsection in the Methods section). Using 5-fold cross-validation, SUMOsu achieved a prediction accuracy, specificity, sensitivity, and Matthew's correlation coefficient of 97%, 98%, 73% and 0.66, respectively. Prediction performance has also been assessed with 10-fold cross validation and self consistency test with a reference of a regular expressions scan (Table 3, details of regular expressions scan can be found in the next subsection). Consistent high MCC values indicated the robustness of this new developed method. However, MCC values may be affected by imbalance of specificity and sensitivity. Therefore, we have investigated the area under ROC curve (AUC), which is not affected by on the imbalance of class distributions. Average AUC of 25 repeats for both 5-fold and 10-fold cross-validation was shown to be as 0.91 (Figure 3). ROC curves and consistent high AUC indicated that, SUMOsu, is actually a stable prediction method.

**Table 3:** Prediction performance of self-consistency, 5-fold cross validation and 10-fold cross-validation tests on the training set.

Evaluation Method	Accuracy	Specificity	Sensitivity	MCC
Self Consistency	0.97	0.98	0.76	0.68
5-fold Cross-validation	0.97	0.98	0.73	0.66
10-fold Cross-validation	0.97	0.98	0.73	0.66
Regular Expressions	0.96	0.97	0.72	0.58

An average of 25 repeats are reported for 5-fold and 10-fold cross validation tests. Standard deviations were less than 0.01, so they were not reported. Regular expressions scan is done by searching [IVLMAP]K.[DE] pattern in the sequence window centering the lysine residue.

We further examined the performance of our prediction method for predicting non-consensus sites of sumoylation. Self-consistency results indicated that our method was able to predict about 12% (11/90) of non-consensus sumoylation sites correctly; however, it was not able to predict any of the non-consensus sumoylation sites in the test data (0/7). On the other hand, prediction accuracy of negative sites following consensus motifs was higher for the training set (~46%, 128/280) and test set (~36%, 8/22). These results also support the results stated in Table 3 and they show that our method outperforms a simple regular expression matching of the ΨKxE/D motif. However, it also gives rise to the question of how these non-consensus sites are processed in the sumoylation pathway and how the prediction of this non-consensus sites can be further improved.

Additionally, in order to assess the contribution of conformational flexibility and disorder to the prediction performance, we have performed 5-fold cross validation and self-consistency tests with and without these features (Table 4). Results showed a slight decrease in sensitivity only. This may be the result of dependence of sequence-based prediction of conformational flexibility and disorder, which may already be included in the sequence based features.

**Table 4:** Effect of conformational flexibility and disorder on prediction performance.

	Self-consistency				5-fold Cross Validation			
**Information**	**Acc**	**Sp**	**Sn**	**MCC**	**Acc**	**Sp**	**Sn**	**MCC**

All Features	0.97	0.98	0.76	0.68	0.97	0.98	0.73	0.66
without Flexibility	0.97	0.98	0.75	0.67	0.97	0.98	0.72	0.66
without Disorder	0.97	0.98	0.75	0.67	0.97	0.98	0.72	0.66
without Flexibility & Disorder	0.97	0.98	0.74	0.67	0.97	0.98	0.72	0.66

Conformational flexibility and disorder features ('DisorderBinary', 'DisorderReal') were eliminated from dataset one by one and together. Self-consistency and 5-fold cross validation tests were performed. An average of 25 repeats are reported for 5-fold cross validation tests. Standard deviations were less than 0.01, so they were not reported.

Overall, the results showed that SUMOsu was able to provide a robust prediction performance showed by different assessment strategies.

### Comparison with other methods

Our proposed method, SUMOsu, has been extensively benchmarked against a set of existing methods: SUMOpre [16], SUMOhydro [23], seeSUMO [22], and SUMOsp2.0 [20]. SUMOsu implements a machine learning approach to sumoylation site prediction, similar to seeSUMO and SUMOhydro. However, it is the first method that employs conformational flexibility, disorder and amino acid volume information in prediction, which may have an impact on sumoylation tendency. We have also compared our proposed method with a simple regular expressions scan of the consensus motif, [IVLMAP]K.[DE].

Initially, we have compared our method with a regular expressions scan. Since there is no chance of performing a cross-validation analysis with motif matching, we choose self-consistency as a comparison strategy. While our self-consistency results were 97%, 98% 75%, 0.70 for accuracy, specificity, sensitivity and MCC respectively, regular expressions scan resulted with 96%, 97%, 72%, 0.58 for the same evaluation measures respectively. Our method showed better results in all of the evaluation measures, and significantly exceeded MCC of a simple regular expressions scan for the training data. For the independent test data, when we set SUMOsu (Threshold = -0.4) sensitivity equal to regular expressions sensitivity value, SUMOsu exceeded regular expressions scan specificity (96%) and accuracy (95%) with a specificity of 97% and an accuracy of 96%. Also, while regular expressions scan resulted with an MCC of 0.56 for test data, SUMOsu resulted with 0.57 for this threshold.

SUMOpre is a statistical method for sumoylation site prediction. It has been trained on a smaller dataset, consisted of 268 positive sites, and 6,361 negative sites. As their web server is not available as of publication date of this article, we have only used measures published in their article to compare with our model. When self-consistency was used as a testing strategy, SUMOpre resulted with 97%, 98%, 74%, 0.64 for accuracy, specificity, sensitivity, and MCC respectively. On the other hand, our method performed better in terms of sensitivity (76%) and MCC (0.68) with a higher dataset of 357 positive sites and 7,909 negative sites.

We have tested our model using an independent dataset in order to compare with other existing methods. We have employed the same independent set as SUMOhydro, which has stated that this set does not contain any residues that have used for any of the training sets of previous methods. In comparison, we have set three thresholds on SVM decision values as previous methods did: low, medium and high. These thresholds has been set as -0.5, 0, and 0.5, respectively. SVM decision values represent distance of the samples to the separating hyperplane. Higher decision values imply deeper points or more confident decisions.

As shown in Table 5 SUMOsu showed the best performance on accuracy and specificity for all thresholds, while seeSUMO-RF exceeded its performance on sensitivity for the medium and high thresholds and SUMOsp2.0 for the low threshold. However, when we set the sensitivity of SUMOsu (Threshold = -0.8) equal to the SUMOsp2.0 sensitivity for the low threshold, we have observed an accuracy of 94%, a specificity of 95% and a MCC of 0.51, indicating a better accuracy, specificity and MCC than SUMOsp2.0 at the same sensitivity level. Additionally, if we set the sensitivity of SUMOsu (Thresholds -0.6 and -0.4 for the medium and high thresholds, respectively) equal to the sensitivity of seeSUMO for the medium and high thresholds, SUMOsu exceeds seeSUMO performance in three evaluation measures with an accuracy of 96%, a specificity of 97%, and a MCC of 0.57; and an accuracy of 96%, a specificity of 97% and a MCC of 0.58 for the medium and low thresholds respectively.

**Table 5:** Comparison of SUMOsu with other predictors.

Method	Threshold	Acc	Sp	Sn	MCC
SUMOsp2.0	*Low*	0.83	0.83	**0.75**	0.30
SUMOhydro		0.91	0.91	0.71	0.41
seeSUMO-RF		0.82	0.83	**0.75**	0.30
seeSUMO-SVM		0.90	0.91	0.67	0.37
**SUMOsu**		**0.96**	**0.97**	0.67	**0.56**
SUMOsp2.0	*Medium*	0.91	0.93	0.63	0.38
SUMOhydro		0.92	0.94	0.67	0.43
seeSUMO-RF		0.88	0.88	**0.71**	0.35
seeSUMO-SVM		0.93	0.95	0.54	0.40
**SUMOsu**		**0.96**	**0.97**	0.58	**0.52**
SUMOsp2.0	*High*	0.95	0.96	0.58	0.47
SUMOhydro		0.93	0.95	0.58	0.42
seeSUMO-RF		0.89	0.90	**0.67**	0.36
seeSUMO-SVM		0.95	0.98	0.38	0.39
**SUMOsu**		**0.96**	**0.98**	0.58	**0.54**
Regular Expressions	*N/A*	0.95	0.96	0.71	0.56

The values of accuracy (Acc), specificity (Sp), sensitivity (Sn), and Matthew's correlation efficients (MCC) are obtained from Chen et al. [23] as the exact same independent dataset was employed in this study. Thresholds for SUMOsu was set as -0.5, 0, and 0.5 for low, medium and high, respectively.

As it can be seen from the aforementioned comparisons, threshold selection significantly affects the performance of SUMOsu in the independent test set and it seems that SUMOsu favors the negative predictions due to class imbalance, which can be solved with appropriate thresholds. All these results show that the proposed method, SUMOsu, compares favorably to the most used sumoylation predictors.

## Discussions

In the present study, we have developed a highly robust new method to predict sumoylation sites with the use of various sequence features and flexibility/disorder information. In order to get rid of redundant features, we have performed a feature selection procedure using RELIEFF [26]. We have also analyzed the data to identify statistically sound differences between positive and negative sites, revealing various amino acid preferences and biochemical differences. Particularly, our study included conformational flexibility and disorder for the first time to our knowledge in sumoylation site prediction, and we have found that these properties statistically significantly differ in positive and negative sites (p < 0.001 for both flexibility and disorder). Although they are not highly effective in improving classification performance, this finding supports the argument of Macauley et al. [27] as conformational flexibility may be a general feature of the regions in sumoylation target lysines. They have summarized that some of the characterized sumoylation sites, such as in histone H4, IκBα, p53, and c-Jun, are located in unstructured flexible regions [27]. However, they have also included some exceptions to this flexibility argument, such as well structured PNT domain of Tel and the RING domain of PML [27]. Similarly, Lin et al. argued that SUMO-1 target binding sites are likely to be located in flexible or unstructured regions [28]. Additionally, they have identified an important surface in substrate recognition, adjacent to the conjugation active site on a SUMO E2 enzyme, UBC9, which has also high flexibility in the picosecond to nanosecond and microsecond to milisecond time scales [28]. When these findings are considered together, it can be argued that both the enzyme surface and the target lysine residues should be conformationally flexible enough to interact and facilitate sumoylation. In fact, it has been shown that the target lysine residue needs to reach into catalytic pocket of UBC9 [29], which may suggest the importance of flexibility in target lysine residue motion. Hence, in the light of specific examples, it can be argued that conformational flexibility or disorder may also be effective in general identification of sumoylation sites. Additionally, it should be noted for disorder that our analyses indicated that consensus positive sites are more disordered than non-consensus positive sites (p = 0.007). This fact may be useful for distinguishing consensus negative sites from positive ones. Ultimately, there may be not be a universal mechanism for sumoylation but instead several distinct mechanisms are in place for sumoylation. Therefore, clustering of sumoylated sites depending on the flexibility of the site and deriving new features to develop separate classifiers for different clusters may better explain the mechanism, which will be the primary focus of a future study by our group.

In addition to conformational flexibility and disorder, one of the main reasons of the robustness of SUMOsu was the employment of sequence information, hydrophobicity information, and estimated amino acid volumes. Effectiveness of hydrophobicity along with position-specific sequence information has been shown in various studies before [23,30]. Our study confirmed these findings and supports Xu et al. [16] and others in the argument that sumoylation significantly depends on local sequence composition. Incorporating hydrophobicity information is also biologically sensible, as hydrophobic patches may be effective in sumoylation site recognition as they were in non-covalent sumo interactions (reviewed in [1]). Similar to previous predictors, we have observed that most of the prediction accuracy is provided by the primary sequence and hydrophobicity information. Also, estimated sub-window volumes may indicate "accessibility" of the central lysine to recognition. They may also represent a degree of mechanistic preferences of sumoylation enzymes while approaching to a protein for target recognition.

## Conclusions

We have developed a new method to predict sumoylation sites by employing support vector machines, which has shown its performance with MCC, sensitivity, specificity, and accuracy equal to 0.66, 73%, 98%, and 97%, respectively for 5-fold cross-validation. We have also performed statistical analyses to identify possible amino acid preferences or other biological factors that may affect the sumoylation site recognition. Our prediction method suggests a sequence and hydrophobicity-dependent recognition affected by conformational flexibility, disorder, or sub-window volume difference. Future work lays in developing a web service for SUMOsu to make it accessible to the biological research community.

## Methods

### Datasets

In the present study, we have used the dataset published by SUMOhydro [23], which contains 382 experimentally validated sumoylation sites in 233 proteins from SUMOsp2.0 [20] dataset and research articles published prior to January 1, 2012. 4 proteins were subtracted from dataset as they do not contain any positive instances. Also, one site has been discarded as the sequence of the referred protein has changed and given position does not contain a lysine residue. Uniprot IDs have been updated for 9 proteins. Lastly, sequences have been updated for 13 proteins since single amino acid substitutions or large deletions have been recorded since SUMOhydro published the dataset, resulting in loss of 2 negative sites and a positive site. A document describing how to update SUMOhydro dataset with stated changes can be found in additional file 3.

In summary, the modified training dataset consisted of 7,909 instead of 8,071 negative sites and 357 instead of 358 positive sites. The independent test set was left untouched as no update was required for those sequences. The test set consisted of 24 positive sites and 510 negative sites.

### Features

The dataset was represented in windows that have experimentally optimized length of 7 (w_c_=3, w_n_=3) (data not shown). Each position of the window sequence (excluding central lysine) was represented with a vector containing only a value of 1 at one of twenty positions representing the amino acids. This sequence encoding resulted in 120 features. Positional naming of amino acids in a window can be found in Figure 2a. For instance, an alanine residue appearing just before the central lysine would be named w-A_3, while a proline residue appearing two residues after central lysine would be named w+P_2. Their hydrophobicity values according to Hopp & Woods hydrophobicity scale [31] were added, forming additional 6 real-valued features. These features are represented with a '-Hydro' tag at the end of the window position. A binary feature representing whether the window conforms to the consensus site was also used. In order to identify whether a negative charge is present in the window, a position-unspecific binary feature (wDE) was used to detect if aspartic acid or glutamic acid was present in the window, regardless of whether they conform to the consensus motif or not. Also, a position-unspecific lysine presence feature (wK) was constructed to account for possible extra positive charges or overlapping sumoylation sites. Conformationally flexible residues were predicted using FlexPred [32] with default parameters and PSSM-based encoding. The status of central lysine according to this prediction was encoded using a single binary feature (Flexible), containing 1 for conformationally flexible, 0 for rigid prediction. Disorder predictions were performed using IUPred [33] and results were represented with one real valued feature (DisorderReal) representing disorder tendency and one binary feature (DisorderBinary) created with a cutoff of 0.5 on predicted disorder tendency value. A binary feature was created to represent whether central lysine is located within 10% of the C- or N-terminals ('terminal regions'). In order to incorporate protein size as well, a real valued feature containing the protein amino acid length multiplied by -1 if the site is in one of the terminal regions, or by +1 if the site is not in any of the terminal regions was encoded in the dataset. Lastly, we have estimated a before central lysine sub-window volume and after central lysine sub-window volume using Kharakoz's amino acid volumes [34], forming 2 additional features (BeforeVol and AfterVol). We have also added the volume difference between sub-windows (negative sub-window volume was subtracted from positive sub-window volume) as a separate feature (Difference).

In total, both training and test sets have been encoded using 137 features: 126 binary and 11 real valued. The encoded training dataset was scaled between 0 and 1 and using the parameters used for training dataset scaling, the test set was also scaled, as it was strongly suggested for kernel-based SVM learning. Explanations of selected features can be found in additional file 4.

### Statistical analysis

The statistical analysis was performed using an in-house program written in Python 2.7.5 [35], with the SciPy [36] library (version 0.11.0). A chi-square test of independence or Mann-Whitney U test was performed to examine the relationship between sumoylated and non-sumoylated sequence windows. For continuous features, this relation was analyzed using two-tailed Mann-Whitney U test, while for binary features chi-square test of independence was used. Yates' correction was only applied for contingency tables containing a cell with a value less than 5 [37]. All cases where Yates' correction was applied has been reported accordingly.

Probability of superiority for Mann-Whitney U test was calculated with following formula:

(1) $$PS=\frac{U}{n_{1}*n_{2}}$$

where, U is the test statistic, and is the sample sizes of positive and negative classes. Also, z-value for Mann-Whitney U test was calculated using with following formula:

(1a) $$z=\frac{U-\frac{n_{1}*n_{2}}{2}}{\sqrt{\frac{n_{1}*n_{2}*\left(n_{1}+n_{2}+1\right)}{12}}}$$

where, U is the test statistic, and is the sample sizes of positive and negative classes.

Benjamini-Hochberg [38] procedure has been applied for controlling false discovery rate at α = 0.05. All reported p-values have been adjusted according to this procedure.

### Feature selection

Feature selection has been performed using RELIEFF [26] algorithm implemented in Weka [39]. RELIEFF basically evaluates "merit" of each feature according to how well their values distinguish among neighbouring instances by repeatedly sampling an instance from the dataset. The default settings in Weka have been used, except nearest neighbours are weighted by their distance. Using these merit scores and corresponding rankings, the classification model was assessed with increasing number of features and their performance was measured with 10-fold cross validation. The best performing model has been selected according to MCC.

### Support vector machine and parameter optimization

Support vector machine (SVM) is a common machine learning algorithm based on the statistical learning theory. A support vector machine mainly constructs a hyperplane in a high-dimensional space to separate feature vectors belonging to different classes using a maximum margin; hence, it aims to achieve low generalization error. If a support vector machine is used for a linear classification, an n-1 dimensional hyperplane is used, where n represents the number of dimensions of the data. In other cases, SVMs map the original data into a high-dimensional feature space through non-linear mapping functions, in order to optimize class seperation. SVMs can then be used for classification, regression, or other tasks.

LibSVM [40] implementation (version 3.17) of SVM algorithm was used in this work. Grid searches were performed for optimizing C and γ parameters of radial basis function (RBF) SVM using Weka [39]. C parameter was searched from 2^-5 ^to 2^15 ^by doubling in each iteration. Similarly, γ parameter was searched from 2^-15 ^to 2^3 ^with the same approach. In case optimal parameters were found in margins, grid extensions were allowed up to 2^5 ^distant from the original grid margins.

Despite their advantages, SVMs are highly sensitive to class imbalance. Seperating hyperplane may be skewed towards the minority class, which in return cause a degraded performance for the minority class [41]. A second possible explanation would be that the amount of support vectors may be imbalanced. In this case, the neighbourhood of a test instance located close to the boundary is more likely to be dominated by negative support vectors, which makes decision function more likely to classify an instance located close to the boundary as a negative instance [41]. In order to overcome this problem, we have adjusted the class weights when training with LibSVM, which adjusts C parameter of the given class asweight*C. We have used 1:5 as class weights in order to overcome class imbalance without resampling.

### Performance assessment

Performance evaluation was done by calculating most common 4 evaluation measures for classification models. Matthew's correlation coefficient (MCC), sensitivity (Sn), specificity (Sp) and accuracy (Acc) were reported with equations (2-5).

(2) $$MCC=\frac{\left(TP*TN\right)-\left(FN*FP\right)}{\sqrt{\left(TP+FN\right)*\left(TN+FP\right)*\left(TP+FP\right)*\left(TN+FN\right)}}$$

(3) $$Sn=\frac{TP}{TP+FN}$$

(4) $$Sp=\frac{TN}{TN+FP}$$

(5) $$Acc=\frac{TP+TN}{TP+FP+TN+FN}$$

where TP is the number of correctly predicted sumoylated windows; TN, correctly predicted not-sumoylated windows; FP, over-predicted windows, and FN, under-predicted windows. All these measures have values ranging from 0 to 1 and higher value indicates a better prediction performance.

In order to determine training errors, 5-fold and 10-fold cross-validation have been performed. Cross-validation is a statistical evaluation technique for measuring the performance of a predictive model. In *n*-fold cross-validation, entire set is randomly divided into *n *subsets. A single subset of the *n *subsets is retained as the test set and the remaining *n*-1 subsets are used as the training set. This process is repeated for *n *times with each subset used exactly once as the testing set. Each cross-validation analysis have been repeated 25 times and the average is reported in the present study. Since all standard deviations were less than 0.01, they were not reported.

In addition to the cross-validation analysis, we have performed a self-consistency test. A self-consistency test is a method where the performance of a model is evaluated according to the same data used for model training. This kind of test reveals the fitting ability of the data characteristics captured by the model.

Performance assessment was also done with receiver operator characteristic (ROC) analysis. One of the most appealing properties of ROC analysis is that it is insensitive to class distribution, as in sumoylation's case there is an obvious imbalance in the negative to positive ratio. ROC curves plot the true positive rate (i.e. Sensitivity, or, TPR) as a function of the false positive rate (i.e. 1-Specificity, or, FPR). The area under ROC curve (AUC) has been widely used to quantify prediction performance using ROC analysis. The AUC gives a measure of the discriminatory value of a classifier at different operating points. Generally, AUC values approaching 1 are considered good predictive models, while AUC = 0.5 indicates a completely random model.

## List of abbreviations used

SUMO: small ubiquitin-like modifier

SVM: support vector machine

Acc: accuracy

Sp: specificity

Sn: sensitivity

MCC: Matthew's correlation coefficient

ROC: receiver operator characteristics

AUC: area under the ROC curve

## Competing interests

The authors declare that they have no competing interests.

## Authors' contributions

ASY participated in the design of the study, carried out the experiments, performed the statistical analyses, and drafted the manuscript. OUS conceived the study, and participated in its design and coordination, and helped to draft the manuscript. All authors read and approved the final manuscript.

## Supplementary Material

Additional file 1 The complete statistical analysis results (*.pdf). The complete statistical testing results for three testing strategies explained in the main text. The test statistics column contains χ² values for chi-square tests, and U for Mann-Whitney U tests. The descriptive statistics column contains the proportions for positive and negative cases in chi-square tests, while it contains the positive and negative population means and standard deviations for Mann-Whitney U tests. Mann-Whitney U tests have extra columns of Z and probability of superiority (PS) values. Click here for file

Additional file 2 The complete list of selected features (*.pdf). The complete list of selected features are presented with their "merit" scores, RELIEFF ranking, and p-values. Click here for file

Additional file 3 How to update the SUMOhydro dataset (*.pdf). Click here for file

Additional file 4 Codebook (*.pdf). Explanations of selected features used throughout the study with their short names. Click here for file

## Declarations

Publication costs for this article were funded by the corresponding author's personal funds.

This article has been published as part of *BMC Genomics *Volume 15 Supplement 9, 2014: Thirteenth International Conference on Bioinformatics (InCoB2014): Computational Biology. The full contents of the supplement are available online at http://www.biomedcentral.com/bmcgenomics/supplements/15/S9.
