# Supplementary material for: Predicting sumoylation sites using support vector machines based on various sequence features, conformational flexibility and disorder
Source: BMC Genomics. 2014 Dec 8;15(Suppl 9):S18. doi: 10.1186/1471-2164-15-S9-S18 (PMC4290605; doi:10.1186/1471-2164-15-S9-S18)
Supplement: Additional file 1 — The complete statistical analysis results (*.pdf). The complete statistical testing results for three testing strategies explained in the main text. The test statistics column contains χ² values for chi-square tests, and U for Mann-Whitney U tests. The descriptive statistics column contains the proportions for positive and negative cases in chi-square tests, while it contains the positive and negative population means and standard deviations for Mann-Whitney U tests. Mann-Whitney U tests have extra columns of Z and probability of superiority (PS) values. [file 1471-2164-15-S9-S18-S3.pdf]

Additional File 3. How to update the SUMOhydro dataset

A patch has been created in order to convert dataset of SUMOhydro to the dataset employed in the present study.

1. You should download the entire dataset as an archive from SUMOhydro supplementary material (http://bit.ly/1gr9z1j), and unpack into a directory.
2. Download the patch file to the same directory from http://bit.ly/sumosupatch
3. For Linux/Unix and Mac OSX systems, use "*patch < Additional_File_1.patch*" command to patch files.
4. If you are using a Windows system, you can download Patch for Windows and use "*patch < Additional_File_1.patch --binary*" command in the Command Prompt.
