# Supplementary material for: Predicting sumoylation sites using support vector machines based on various sequence features, conformational flexibility and disorder
Source: BMC Genomics. 2014 Dec 8;15(Suppl 9):S18. doi: 10.1186/1471-2164-15-S9-S18 (PMC4290605; doi:10.1186/1471-2164-15-S9-S18)
Supplement: Additional file 2 — The complete list of selected features (*.pdf). The complete list of selected features are presented with their "merit" scores, RELIEFF ranking, and p-values. [file 1471-2164-15-S9-S18-S2.pdf]

| Rank | Feature | Merit Score | P-value | Adjusted P-value | Significance |
|---|---|---|---|---|---|
| 1 | w+E_2 | 0.355474 | 0.00E+00 | 0.00E+00 | * |
| 2 | Consensus | 0.261813 | 0.00E+00 | 0.00E+00 | * |
| 3 | wDE | 0.164459 | 1.66E-41 | 3.23E-40 | * |
| 4 | w+2_Hydro | 0.160149 | 3.90E-83 | 1.33E-81 | * |
| 5 | w-I_3 | 0.105916 | 1.18E-107 | 5.33E-106 | * |
| 6 | w-3_Hydro | 0.104835 | 8.12E-58 | 1.84E-56 | * |
| 7 | wK | 0.078651 | 1.33E-02 | 4.03E-02 | * |
| 8 | w-V_3 | 0.075073 | 1.92E-58 | 5.22E-57 | * |
| 9 | w-2_Hydro | 0.057669 | 1.48E-02 | 4.37E-02 | * |
| 10 | w+3_Hydro | 0.056496 | 1.49E-01 | 2.93E-01 | |
| 11 | w+1_Hydro | 0.05232 | 7.13E-02 | 1.62E-01 | |
| 12 | w-1_Hydro | 0.051279 | 4.22E-02 | 9.89E-02 | |
| 13 | w-L_3 | 0.051001 | 1.96E-03 | 7.00E-03 | * |
| 14 | w+K_2 | 0.050248 | 1.70E-08 | 1.78E-07 | * |
| 15 | w+P_2 | 0.045911 | 3.39E-02 | 8.23E-02 | |
| 16 | w+P_3 | 0.043573 | 2.11E-25 | 3.58E-24 | * |
| 17 | w-K_3 | 0.043208 | 4.33E-04 | 1.96E-03 | * |
| 18 | Flexibility | 0.042334 | 7.52E-07 | 7.31E-06 | * |
| 19 | w+D_2 | 0.041784 | 7.14E-01 | 7.77E-01 | |
| 20 | w-S_2 | 0.041097 | 2.95E-01 | 4.57E-01 | |
| 21 | DisorderBinary | 0.040666 | 7.27E-14 | 9.88E-13 | * |
| 22 | w-E_3 | 0.039548 | 6.14E-05 | 3.79E-04 | * |
| 23 | w-A_3 | 0.03804 | 3.04E-02 | 7.95E-02 | |
| 24 | w-P_1 | 0.037893 | 4.46E-06 | 3.80E-05 | * |
| 25 | w-E_2 | 0.035935 | 4.16E-01 | 5.74E-01 | |
| 26 | w-Q_2 | 0.035399 | 3.27E-02 | 8.10E-02 | |
| 27 | w-K_1 | 0.035294 | 5.88E-01 | 7.27E-01 | |
| 28 | w+E_3 | 0.035173 | 5.54E-01 | 7.24E-01 | |
| 29 | w+E_1 | 0.034129 | 1.08E-03 | 4.37E-03 | * |
| 30 | w-L_2 | 0.033322 | 1.53E-01 | 2.95E-01 | |
| 31 | w+G_2 | 0.031063 | 1.64E-03 | 6.21E-03 | * |
| 32 | termini | 0.030254 | 2.16E-01 | 3.74E-01 | |
| 33 | w-P_3 | 0.028768 | 4.81E-01 | 6.47E-01 | |
| 34 | w+S_2 | 0.0276 | 1.73E-04 | 9.07E-04 | * |
| 35 | w+K_1 | 0.027429 | 7.28E-01 | 7.85E-01 | |
| 36 | w+K_3 | 0.0272 | 8.18E-01 | 8.62E-01 | |
| 37 | w-L_1 | 0.027056 | 2.99E-01 | 4.57E-01 | |
| 38 | w-E_1 | 0.026271 | 1.43E-03 | 5.54E-03 | * |
| 39 | w+L_1 | 0.026226 | 2.27E-01 | 3.82E-01 | |
| 40 | BeforeVol | 0.025254 | 2.58E-02 | 6.89E-02 | |
| 41 | w+L_2 | 0.025245 | 9.00E-09 | 1.02E-07 | * |
| 42 | w-S_1 | 0.025126 | 6.47E-01 | 7.52E-01 | |

| | | | | | |
|---|---|---|---|---|---|
| 43 | w-D_2 | 0.023201 | 9.58E-01 | 9.80E-01 | |
| 44 | w-S_3 | 0.022316 | 1.30E-06 | 1.18E-05 | * |
| 45 | w+V_1 | 0.02217 | 5.19E-01 | 6.86E-01 | |
| 46 | w-V_1 | 0.021961 | 5.87E-05 | 3.79E-04 | * |
| 47 | w+P_1 | 0.021959 | 3.98E-01 | 5.58E-01 | |
| 48 | w-Q_1 | 0.02193 | 6.31E-01 | 7.52E-01 | |
| 49 | w+A_1 | 0.020875 | 1.46E-01 | 2.93E-01 | |
| 50 | w-R_3 | 0.020717 | 2.10E-05 | 1.59E-04 | * |
| 51 | w+D_1 | 0.020657 | 1.81E-02 | 5.13E-02 | |
| 52 | w+Q_3 | 0.020378 | 5.77E-01 | 7.27E-01 | |
| 53 | w-K_2 | 0.02033 | 8.17E-02 | 1.76E-01 | |
| 54 | w+V_3 | 0.019885 | 2.42E-01 | 3.97E-01 | |
| 55 | DisorderReal | 0.019499 | 2.05E-14 | 3.10E-13 | * |
| 56 | w+T_3 | 0.01926 | 8.81E-01 | 9.08E-01 | |
| 57 | w-G_2 | 0.019222 | 5.64E-02 | 1.30E-01 | |
| 58 | w-T_1 | 0.018905 | 9.94E-02 | 2.08E-01 | |
| 59 | w+M_1 | 0.018893 | 5.26E-03 | 1.74E-02 | * |
| 60 | w+R_2 | 0.018604 | 1.34E-04 | 7.59E-04 | * |
| 61 | w+S_3 | 0.018554 | 9.73E-01 | 9.88E-01 | |
| 62 | w-R_1 | 0.018388 | 4.45E-01 | 6.05E-01 | |
| 63 | w+F_3 | 0.018034 | 4.18E-01 | 5.74E-01 | |
| 64 | w+N_1 | 0.017975 | 1.56E-01 | 2.95E-01 | |
| 65 | w-D_1 | 0.017962 | 7.62E-02 | 1.67E-01 | |
| 66 | w-F_2 | 0.017845 | 7.10E-01 | 7.77E-01 | |
| 67 | w-F_3 | 0.017749 | 5.73E-01 | 7.27E-01 | |
| 68 | w+I_3 | 0.017586 | 1.93E-01 | 3.45E-01 | |
| 69 | w+R_3 | 0.016933 | 1.19E-01 | 2.46E-01 | |
| 70 | w+R_1 | 0.016767 | 9.17E-02 | 1.95E-01 | |
| 71 | w+Q_1 | 0.016723 | 7.48E-13 | 9.25E-12 | * |
| 72 | w-I_1 | 0.016717 | 3.66E-01 | 5.30E-01 | |
| 73 | w+F_1 | 0.016368 | 6.69E-01 | 7.58E-01 | |
| 74 | AfterVol | 0.016063 | 1.07E-02 | 3.39E-02 | * |
| 75 | w-P_2 | 0.015921 | 7.40E-01 | 7.92E-01 | |
| 76 | w+L_3 | 0.015276 | 7.57E-02 | 1.67E-01 | |
| 77 | w-V_2 | 0.014765 | 1.83E-01 | 3.32E-01 | |
| 78 | w+F_2 | 0.01459 | 1.62E-02 | 4.70E-02 | * |
| 79 | w-G_1 | 0.014474 | 3.76E-01 | 5.33E-01 | |
| 80 | w-R_2 | 0.014341 | 6.36E-01 | 7.52E-01 | |
| 81 | w+C_1 | 0.013886 | 3.37E-01 | 4.98E-01 | |
| 82 | w-A_1 | 0.013816 | 9.89E-01 | 9.97E-01 | |
| 83 | w+T_1 | 0.012826 | 1.02E-05 | 8.15E-05 | * |
| 84 | Difference | 0.01279 | 5.02E-04 | 2.13E-03 | * |
| 85 | w+D_3 | 0.012263 | 6.31E-01 | 7.52E-01 | |

| | | | | | |
|---|---|---|---|---|---|
| 86 | w+M_3 | 0.011948 | 5.88E-01 | 7.27E-01 | |
| 87 | w-D_3 | 0.011607 | 1.86E-04 | 9.37E-04 | * |
| 88 | w+S_1 | 0.011454 | 6.95E-01 | 7.69E-01 | |
| 89 | w-T_3 | 0.011305 | 3.27E-03 | 1.11E-02 | * |
| 90 | w+V_2 | 0.011231 | 4.97E-04 | 2.13E-03 | * |
| 91 | w-I_2 | 0.011083 | 2.65E-01 | 4.20E-01 | |
| 92 | w+A_3 | 0.01096 | 1.79E-01 | 3.29E-01 | |
| 93 | w-G_3 | 0.010915 | 1.73E-04 | 9.07E-04 | * |
