# Supplementary material for: Predicting sumoylation sites using support vector machines based on various sequence features, conformational flexibility and disorder
Source: BMC Genomics. 2014 Dec 8;15(Suppl 9):S18. doi: 10.1186/1471-2164-15-S9-S18 (PMC4290605; doi:10.1186/1471-2164-15-S9-S18)
Supplement: Additional file 3 — How to update the SUMOhydro dataset (*.pdf). [file 1471-2164-15-S9-S18-S1.pdf]

Additional File 1. How to update the SUMOhydro dataset

A patch has been created in order to convert dataset of SUMOhydro to the dataset employed in the present study.

1. You should download the entire dataset as an archive from SUMOhydro supplementary material (http://bit.ly/1gr9z1j), and unpack into a directory.
2. Download the patch file to the same directory from http://bit.ly/sumosupatch
3. For Linux/Unix and Mac OSX systems, use "*patch < Additional_File_1.patch*" command to patch files.
4. If you are using a Windows system, you can download Patch for Windows and use "*patch < Additional_File_1.patch --binary*" command in the Command Prompt.
