# Supplementary material for: Predicting sumoylation sites using support vector machines based on various sequence features, conformational flexibility and disorder
Source: BMC Genomics. 2014 Dec 8;15(Suppl 9):S18. doi: 10.1186/1471-2164-15-S9-S18 (PMC4290605; doi:10.1186/1471-2164-15-S9-S18)
Supplement: Additional file 4 — Codebook (*.pdf). Explanations of selected features used throughout the study with their short names. [file 1471-2164-15-S9-S18-S4.pdf]

Feature	Range	Explanation
w+E_2	0 or 1	Presence of amino acid E, 2nd position after central lysine
Consensus	0 or 1	Presence of consensus motif [IVLMAP]K.[DE]
wDE	0 or 1	Presence of D or E amino acids in the sequence window, position unspecific
w+2_Hydro	0 - 1 (Scaled)	Hopp & Woods Hydrophobicity value of the amino acid, 2nd position after central lysine
w-I_3	0 or 1	Presence of amino acid I, right before central lysine
w-3_Hydro	0 - 1 (Scaled)	Hopp & Woods Hydrophobicity value of the amino acid, right before central lysine
wK	0 or 1	Presence of K amino acid, apart from the central lysine, position unspecific
w-V_3	0 or 1	Presence of amino acid V, right before central lysine
w-2_Hydro	0 - 1 (Scaled)	Hopp & Woods Hydrophobicity value of the amino acid, 2 amino acids before central lysine
w+3_Hydro	0 - 1 (Scaled)	Hopp & Woods Hydrophobicity value of the amino acid, 3rd position after central lysine
w+1_Hydro	0 - 1 (Scaled)	Hopp & Woods Hydrophobicity value of the amino acid, 1st position after central lysine
w-1_Hydro	0 - 1 (Scaled)	Hopp & Woods Hydrophobicity value of the amino acid, 3 amino acids before central lysine
w-L_3	0 or 1	Presence of amino acid L, right before central lysine
w+K_2	0 or 1	Presence of amino acid K, 2nd position after central lysine
w+P_2	0 or 1	Presence of amino acid P, 2nd position after central lysine
w+P_3	0 or 1	Presence of amino acid P, 3rd position after central lysine
w-K_3	0 or 1	Presence of amino acid K, right before central lysine
Flexibility	0 or 1	Flexibility of central lysine residue
w+D_2	0 or 1	Presence of amino acid D, 2nd position after central lysine
w-S_2	0 or 1	Presence of amino acid S, 2 amino acids before central lysine
DisorderBinary	0 or 1	Disorder tendency of central lysine, predicted by IUPred (cutoff = 0.5)
w-E_3	0 or 1	Presence of amino acid E, right before central lysine
w-A_3	0 or 1	Presence of amino acid A, right before central lysine
w-P_1	0 or 1	Presence of amino acid P, 3 amino acids before central lysine
w-E_2	0 or 1	Presence of amino acid E, 2 amino acids before central lysine
w-Q_2	0 or 1	Presence of amino acid Q, 2 amino acids before central lysine
w-K_1	0 or 1	Presence of amino acid K, 3 amino acids before central lysine

w+E_3	0 or 1	Presence of amino acid E, 3rd position after central lysine
w+E_1	0 or 1	Presence of amino acid E, 1st position after central lysine
w-L_2	0 or 1	Presence of amino acid L, 2 amino acids before central lysine
w+G_2	0 or 1	Presence of amino acid G, 2nd position after central lysine
termini	0 or 1	Whether central lysine is located in the 10% of N- or C- terminal of the protein
w-P_3	0 or 1	Presence of amino acid P, right before the central lysine
w+S_2	0 or 1	Presence of amino acid S, 2nd position after central lysine
w+K_1	0 or 1	Presence of amino acid K, 1st position after central lysine
w+K_3	0 or 1	Presence of amino acid K, 3rd position after central lysine
w-L_1	0 or 1	Presence of amino acid L, 3 amino acids before central lysine
w-E_1	0 or 1	Presence of amino acid E, 3 amino acids before central lysine
w+L_1	0 or 1	Presence of amino acid L, 1st position after central lysine
BeforeVol	0 - 1 (Scaled)	Sum of amino acid volumes before central lysine
w+L_2	0 or 1	Presence of amino acid L, 2nd position after central lysine
w-S_1	0 or 1	Presence of amino acid S, 3 amino acids before central lysine
w-D_2	0 or 1	Presence of amino acid D, 2 amino acids before central lysine
w-S_3	0 or 1	Presence of amino acid S, right before central lysine
w+V_1	0 or 1	Presence of amino acid V, 1st position after central lysine
w-V_1	0 or 1	Presence of amino acid V, 3 amino acids before central lysine
w+P_1	0 or 1	Presence of amino acid P, 1st position after central lysine
w-Q_1	0 or 1	Presence of amino acid Q, 3 amino acids before central lysine
w+A_1	0 or 1	Presence of amino acid A, 1st position after central lysine
w-R_3	0 or 1	Presence of amino acid R, right before central lysine
w+D_1	0 or 1	Presence of amino acid D, 1st position after central lysine
w+Q_3	0 or 1	Presence of amino acid Q, 3rd position after central lysine
w-K_2	0 or 1	Presence of amino acid K, 2 amino acids before central lysine
w+V_3	0 or 1	Presence of amino acid V, 3rd position after central lysine
DisorderReal	0 - 1	Disorder tendency of central lysine, predicted by IUPred

w+T_3	0 or 1	Presence of amino acid T, 3rd position after central lysine
w-G_2	0 or 1	Presence of amino acid G, 2 amino acids before central lysine
w-T_1	0 or 1	Presence of amino acid T, 3 amino acids before central lysine
w+M_1	0 or 1	Presence of amino acid M, 1st position after central lysine
w+R_2	0 or 1	Presence of amino acid R, 2nd position after central lysine
w+S_3	0 or 1	Presence of amino acid S, 3rd position after central lysine
w-R_1	0 or 1	Presence of amino acid R, 3 amino acids before central lysine
w+F_3	0 or 1	Presence of amino acid F, 3rd position after central lysine
w+N_1	0 or 1	Presence of amino acid N, 1st position after central lysine
w-D_1	0 or 1	Presence of amino acid D, 3 amino acids before central lysine
w-F_2	0 or 1	Presence of amino acid F, 2 amino acids before central lysine
w-F_3	0 or 1	Presence of amino acid F, right before central lysine
w+I_3	0 or 1	Presence of amino acid I, 3rd position after central lysine
w+R_3	0 or 1	Presence of amino acid R, 3rd position after central lysine
w+R_1	0 or 1	Presence of amino acid R, 1st position after central lysine
w+Q_1	0 or 1	Presence of amino acid Q, 1st position after central lysine
w-I_1	0 or 1	Presence of amino acid I, 3 amino acids before central lysine
w+F_1	0 or 1	Presence of amino acid F, 1st position after central lysine
AfterVol	0 - 1 (Scaled)	Sum of amino acid volumes after central lysine
w-P_2	0 or 1	Presence of amino acid P, 2 amino acids before central lysine
w+L_3	0 or 1	Presence of amino acid L, 3rd position after central lysine
w-V_2	0 or 1	Presence of amino acid V, 2 amino acids before central lysine
w+F_2	0 or 1	Presence of amino acid F, 2nd position after central lysine
w-G_1	0 or 1	Presence of amino acid G, 3 amino acids before central lysine
w-R_2	0 or 1	Presence of amino acid R, 2 amino acids before central lysine
w+C_1	0 or 1	Presence of amino acid C, 1st position after central lysine
w-A_1	0 or 1	Presence of amino acid A, 3 amino acids before central lysine
w+T_1	0 or 1	Presence of amino acid T, 1st position after central lysine

Difference	0 - 1 (Scaled)	Difference of BeforeVol and AfterVol features
w+D_3	0 or 1	Presence of amino acid D, 3rd position after central lysine
w+M_3	0 or 1	Presence of amino acid M, 3rd position after central lysine
w-D_3	0 or 1	Presence of amino acid D, right before central lysine
w+S_1	0 or 1	Presence of amino acid S, 1st position after central lysine
w-T_3	0 or 1	Presence of amino acid T, right before central lysine
w+V_2	0 or 1	Presence of amino acid V, 2nd position after central lysine
w-I_2	0 or 1	Presence of amino acid I, 2 amino acids before central lysine
w+A_3	0 or 1	Presence of amino acid A, 3rd position after central lysine
w-G_3	0 or 1	Presence of amino acid G, right before central lysine
